# Prevalence and risk factors for visual impairment among elderly patients attending the eye clinic at Mulago National Referral Hospital, Uganda: a cross-sectional study

**DOI:** 10.4314/ahs.v22i2.18S

**Published:** 2022-08

**Authors:** Ben Mulinde, Immaculate Atukunda, Ismael Kawooya, Francis O Sebabi, David Mukunya, Charles Batte, Damalie Nakanjako, Lydia Nakiyingi, Caroline Nalukenge, Faith Nakubulwa, Anne A Musiika, Juliet Otiti-Sengeri

**Affiliations:** 1 Department of Ophthalmology, School of Medicine, Makerere University College of Health Sciences; 2 Department of Medicine, School of Medicine, Makerere University College of Health Sciences; 3 The Center for Rapid Evidence Synthesis (ACRES), Regional East African Community Health (REACH) Policy Initiative, Makerere University College of health sciences; 4 Department of Community and Public Health, Faculty of health Sciences, Busitema University, Mbale, Uganda; 5 Lung Institute, Department of Medicine, School of Medicine, College of Health Sciences, Makerere University, Kampala, Uganda

**Keywords:** Visual impairment, elderly patients, eye clinic, Mulago National Referral Hospital, Uganda

## Abstract

**Background:**

The elderly have an increased risk of developing visual impairment (VI). Due to the increase in life expectancy of individuals in Sub-Saharan Africa, the population of the elderly is projected to increase. It is thus postulated that the prevalence of VI will increase which is currently unknown in Uganda.

**Objective:**

To determine the prevalence and risk factors for VI among the elderly at Mulago National Referral Hospital eye clinic in Uganda.

**Methods:**

This was a cross-sectional study carried out in 2020 with consecutive enrolment of patients aged 60 years and above. Obtaining history was followed by systemic and ocular examination. Statistical analysis was performed to determine the prevalence and factors associated with VI.

**Results:**

Of 346 elderly participants examined, 174 (50.3%) were males and median age was 67 (IQR 63–74). Prevalence of VI was 32.1%. Cataract was the leading cause of blindness 54.1%, followed by refractive error (21.6%), glaucoma (11.7%), and corneal opacities (5.4%). Age (adjusted Prevalence Ratio (aPR): 1.05, 95% CI (1.02, 1.06)), history of diabetes mellitus (aPR 1.46, 95%CI (1.04, 2.05)), history of hypertension (aPR 1.46, 95%CI (1.10, 1.93)), having completed primary level of education (aPR 0.74, 95%CI (0.55, 0.98)) and secondary level of education (aPR 0.47, 95%CI (0.30,0.73)), presence of a cataract at examination (aPR: 2.28, 95%CI (1.66, 3.13)) were statistically significantly associated with VI.

**Conclusion:**

In Mulago hospital, the prevalence of VI among the elderly is high with majority of the causes being correctable. We recommend that efforts towards early case identification of causes of VI among the elderly should be a priority.

## Introduction

Visual impairment (VI) results in loss of economic and educational opportunities, reduced quality of life, and increased risk of falls and death^1^, ^2^. People with VI suffer significant healthcare costs and reduced quality of life due to increased morbidity and mortality^3^. Globally, VI is caused by common eye diseases, such as glaucoma, cataracts, and age-related macular degeneration- are related to aging.^4^–^6^ thus people aged 50 years and above contribute 82% of the global burden of blindness^1^. In Uganda, the population of older persons (60 and above) is estimated to be 1.6 million and is expected to increase to 5.5 million by 2050^7^. An increase in the elderly population will increase the burden of non-communicable diseases such as blindness^8^.

The Global Eye Health Plan action plan, 2014–2019 stresses the need to undertake epidemiological surveys on VI at regular intervals both nationally and sub-nationally to generate evidence on the magnitude and causes of VI^9^. Unfortunately, there is a scarcity of data on the prevalence and factors associated with VI among the elderly population of Uganda. This makes advocating and developing policies for efficient eye care in this vulnerable population difficult. Therefore, we sought to determine the prevalence of VI and the associated factors among the elderly attending Mulago National Referral Hospital eye clinics in Uganda.

## Material and Methods

### Study Design

This was a cross-sectional study.

### Study setting

This study was conducted among patients aged 60 years and above at Mulago National Referral Hospital in Uganda carried out during February and March 2020. Mulago Hospital is one of the six national referral hospitals in Uganda and has the largest ophthalmology department in terms of staff, equipment, and patient attendance. It also doubles as the teaching hospital for Makerere University. The ophthalmology department has two eye clinics: a screening clinic ran by ophthalmic clinical officers (diploma level) and a consultation clinic ran by ophthalmologists. Both clinics run from Monday to Friday, with an estimated daily elderly patient attendance of 10 due to lockdown restrictions during the COVID-19 pandemic. This is half of the number of elderly patients seen before the pandemic. The study participants were recruited from both eye clinics.

We defined elderly as individuals 60 years and above according to United Nations definition of the elderly^10^. We excluded patients with known allergies to drops (cyclopentolate and amethocaine) or Fluorescein stain used in the study for ocular examination and those who were too sick to withstand the rigor of a full ocular exam were excluded.

### Sample size and sampling of the study population

We consecutively sampled patients attending the eye clinic. We used a sample size of 346 derived using a desired precision of 0.05 and assuming a VI prevalence of 34.2%^11^.

### Data sources/measurement

A pretested structured questionnaire administered by the researcher or trained research assistants (Ophthalmology clinical officers and nurses), was used for data collection. Participants' demographic characteristics, social, ocular, and medical history were collected.

A detailed ocular examination was done by the principal investigator starting with the right eye then the left eye. This included: distance visual acuity using a 6 m Snellen's chart or illiterate E chart; those with visual acuity (V/A) less than 6/6 were reassessed with a pinhole and then refracted with an autorefractometer. Near vision was then assessed using a Jaeger chart, and then refraction was done on all participants with impaired near vision. Visual fields were assessed by the confrontational method compared with the examiner (the examiner had normal visual fields confirmed by perimetry). Extra ocular muscle activity was assessed; the cover-uncover test was done to assess for phoria. Diplopia was sought for in all directions of gaze. Amsler grid was done in all subjects to assess macular function. Examination of the lids, conjunctiva, cornea, anterior chamber, pupil, and iris was done using a slit lamp. Tonometry using Perkin's applanation tonometer, after instilling an anesthetic drop (tetracaine) and staining the tear film with fluorescein strips, was carried out on all respondents. Dilating of the pupil was done using cyclopentolate eye drops and then indirect ophthalmoscopy was performed in study participants with a visual acuity less than 6/6. Investigations were determined on an individual basis to aid in achieving the study objectives and these included optical coherence tomography, fluorescein stain, X-ray of the orbit, ultra sound scan, and computerized tomography.

We defined VI according to the WHO International Classification of Diseases 11 (2018) as presenting visual acuity in the better eye worse than 6/12 in the elderly. VI was further graded as mild, moderate, severe, and blindness according to the extent of visual acuity as follows:

- Mild VI: Presenting visual acuity worse than 6/12 to 6/18
- Moderate VI: Presenting visual acuity worse than 6/18 to 6/60
- Severe VI: Presenting visual acuity worse than 6/60 to 3/60
- Blindness: Presenting visual acuity worse than 3/60

Any ocular anomaly detected during the patient assessment was both documented and managed where possible, or the relevant specialist was consulted on the course of management and referral.

### Statistical analysis

Descriptive statistical measures such as means, standard deviations and medians, interquartile range, frequencies, proportions, and percentages for continuous and categorical variables wherever appropriate, were computed. The prevalence of VI among the elderly attending the eye clinic at Mulago National Referral Hospital was calculated as a proportion of the number of elderly with VI over the total number of elderly enrolled in the study.

In bivariate and multivariate analysis, the modified Poisson regression model with robust variance estimation were used to estimate the prevalence risk ratios and their 95% CI. The outcome was dichotomized as yes = 1, if one had any degree of VI, and no = 0, if one had no VI. A forward stepwise multivariate model was constructed for variables that were significant at p<0.2 during bivariate analysis and those considered clinically significant. All results were considered significant if the P-value was at < 0.05 and a 95% CI that did not cross the null value. Statistical analysis was performed using STATA 15.0 (College Station, Texas, USA).

## Results

### Characteristics of the study population

A total of 346 elderly participated in the study with an equal distribution of the sexes. The median age was 67 years (interquartile range [IQR] of 63–74) with most (57.2%) of the elderly falling in the 60–69 age group. About 4% of the elderly reported a history of trauma while 28% had a history of an eye operation. The commonly reported chronic illnesses were Diabetes (11.3%), hypertension (23.5%), human immunodeficiency virus (HIV) (2.9%). This is summarized in Table 1 below

**Table 1:** Table showing demographic characteristics, social economic characteristics, and history of study participants

Variable	Frequency (%) N=346
**Age (completed years)**	
60–69	198 (57.2)
70–79	98 (28.3)
≥80	50 (14.5)
**Sex**	
Male	174 (50.3)
Female	172 (49.7)
**Highest Level of education**	
None	85 (24.6)
Primary	135 (39.0)
Secondary	99 (28.6)
Tertiary	27 (7.8)
**Marital status**	
Single	11 (3.2)
Married	172 (49.7)
Divorced	34 (9.8)
Widowed	129 (37.3)
**Do you any form of employment/income**	
Yes	157 (45.8)
No	186 (54.2)
**Nature of occupation**	
Business	30 (22.1)
Farming	78 (57.4)
Office related	15 (11.0)
other	13 (9.6)
**Previous form of employment**	
Self	136 (66.3)
Employed	69 (33.7)
**Have you ever smoked**	
Yes	13 (3.8)
No	333 (96.2)
**Do you drink alcohol**	
Yes	28 (8.1)
No	318 (91.9)
**Have you ever had eye trauma**	
Yes	15 (4.4)
No	323 (95.3)
**Have you ever had an eye operation**	
Yes	97 (28)
No	248 (72)
**Do you use visual correction**	
Yes	47 (13.6)
No	299 (86.4)
**History of Diabetes**	
Yes	39 (11.3)
No	307 (88.7)
**History of Hypertension**	
Yes	81 (23.5)
No	265 (76.5)
**Reported HIV status**	
Positive	10 (2.9)
Negative	336 (97.1)
**History of any other chronic illness** *	
Yes	10 (2.9)
No	336 (97.1)

* Other chronic illnesses included asthma, arthritis, peptic ulcer disease, cancers of the thyroid, breast, and cervix.

The most common ocular morbidities were; cataracts (24.82%), refractive error (22.61%), pseudophakia (13.42%) as summarized in Table 2 below.

**Table 2:** Ocular disorders among study participants

Diagnosis	Frequency (%)
Mild Visual Impairment	29 (8.4)
Moderate visual impairment	51 (14.7)
Severe visual impairment	6 (1.7)
Blindness	25 (7.2)
Cataracts	135 (24.8)
Refractive errors	123(22.6)
Pseudophakia	73 (13.4)
Glaucoma	27(5)
Allergic conjunctivitis	27(5)
Bacterial conjunctivitis	17 (3.1)
Pterygium	12 (2.2)
Corneal opacity	4 (0.7)
Dry eye syndrome	4 (0.7)
Others	98 (18)

*Others include: Age related macular degeneration, hypertensive and diabetic retinopathy, uveitis, ocular and orbital tumors

### Prevalence of visual impairment among the elderly

**Figure 1:**
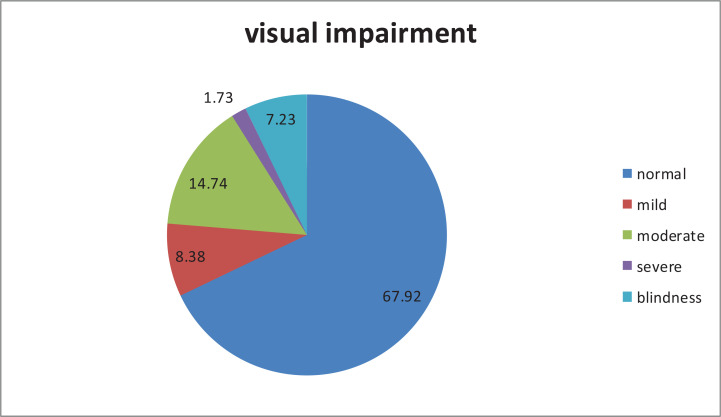
Prevalence of visual impairment among the elderly

Prevalence of VI among the elderly was 32.08%; the different subcategories are shown in Figure 1 below.

The common diseases found among the elderly with VI were cataracts at 54.05%, refractive error at 21.62%, glaucoma at 11.71%, corneal opacities at 5.4%, age-related macular degeneration at 1.8% and hypertensive retinopathy at 1.8%.

**Figure 2:**
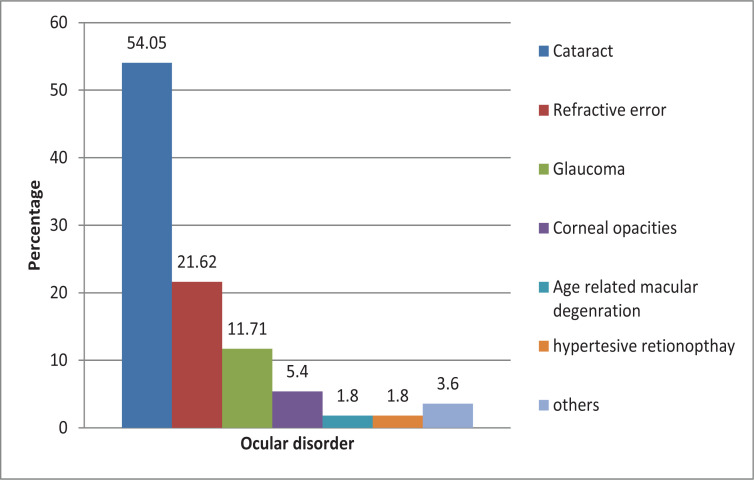
Diseases among the elderly with visual impairment

Other findings (3.6%) were macular hole (0.9%), uveitis (0.9%, corneal ulcers (0.9%), and bullous keratopathy (0.9%) as shown in figure 2 below.

### Factors associated with visual impairment among the elderly

Age, history of diabetes mellitus (DM), history of hypertension, having primary and secondary education, and presence of cataract at examination were statistically significantly associated with VI. This is shown in Table 3 below.

**Table 3:** Bivariate and multivariate analysis for factors associated with visual impairment among the elderly

	Bivariate analyses			Multi-variate model		
	PR	95% CI	P value	PR	95% CI	P value
**Age of the participant**	1.07	1.05, 1.08	0.0001	1.05	1.03, 1.06	<0.001
**Sex of participant**						
Male	1					
Female	0.99	0.73,1.35	0.967			
**Completed level of education**						
None	1			1		
Primary	0.63	0.45,0.87		0.74	0.55,0.98	0.036
Secondary	0.34	0.21,0.55		0.47	0.30,0.73	0.001
University	0.59	0.31,1.10	0.0001	0.71	0.34,1.45	0.341
**Marital status**						
Single	1					
Married	0.34	0.20,0.59				
Divorced	0.51	0.26,1.00				
Widow	0.68	0.41,1.13	0.0001			
**Family history of any ocular disease**						
No	1					
Yes	1.96	1.12,3.41	0.019			
**History of diabetes**						
No	1.00		1			
Yes	1.63	1.12,2.35	0.01	1.46	1.04,2.05	0.029
**History of hypertension**						
No	1.00			1		
Yes	1.83	1.36,2.47	0.001	1.46	1.10,1.93	0.008
**Cataract**						
No	1.00			1		
Yes	2.89	2.08,3.99	0.0001	2.28	1.66,3.13	<0.001
**Refractive error**						
No	1.00					
Yes	0.67	0.46,0.97	0.034			
**Glaucoma**						
No	1.00					
Yes	1.43	0.90,2.26	0.126			
**Pseudophakia**						
No	1.00					
Yes	1.32	0.94,1.85	0.104			

PR: Prevalence ratio, CI confidence interval

*Forms/sources of income included farming, business, office related work, others

With every added year, the prevalence of VI increased by 5%. The odds of VI was 44% higher among elders with a history of DM compared to those without. Those with hypertension had 42% higher odds of having VI compared to those without. The elders who had primary education and those with secondary education were 26% and 53%, respectively, less likely to have VI. Presence of cataracts on examination caused an increase in the odds of having VI by 2.3 times.

## Discussion

We assessed the prevalence and factors associated with visual impairment (VI) among the elderly attending the eye clinic at Mulago National Referral Hospital. About a third of the elderly had VI with half of these having cataracts. Other conditions found in this study in order of frequency were refractive errors, glaucoma, corneal opacities, age-related macular degeneration, and hypertensive retinopathy. Increasing age, history of diabetes mellitus, history of hypertension, and primary and secondary education were significantly associated with VI.

The prevalence found in our study was higher than studies done in Taiwan (17.7%), Delhi (24.5%), and Afghanistan (22.6%)^12^–^14^. However, these studies were population-based studies. Furthermore, with cataracts being the main cause of VI, the cataract surgical rate in these countries is higher than in Uganda^15^, ^16^. In sub Saharan Africa, it is estimated that only one out of ten cataracts ever gets operated 17. The prevalence was comparable to a population-based study done in Nigeria that showed the prevalence of VI among pensioners to be 34.2%^11^.

In this study, presence of cataract at examination, age, history of diabetes mellitus, history of hypertension, and education were statistically significantly associated with VI. Similar to our study, several studies have found cataracts to be associated with VI among elders^1^, ^13^, ^18^–^20^. In low and middle income countries (LMICs), the cataract surgical rate is low thus many people especially the elderly with visually impairing cataracts live with VI as they await cataract surgery^15^, ^16^. This study also found increasing age to be associated with VI which has been documented in several other studies. Increasing age increases ones risk to most of the leading blinding conditions like cataracts, glaucoma, age related macular degeneration and diabetic retinopathy^2^, ^12^, ^21^-^23^. Furthermore, the elders with history of either diabetes or hypertension were more likely to be visually impaired compared to those without. These systemic diseases affect the eyes causing different disorders that affect vision like early onset of cataracts, diabetic retinopathy, hypertensive retinopathy, retinal vascular occlusions^24^–^26^. Studies done among the elderly in other LMICs have reported similar findings^21^, ^22^. However, the study participants who were educated were less likely to have VI which is similar to the findings from a study done in Northern Indian^27^. It has been noted that VI prevention and its correction are not frequent in subjects with low education levels and there is also poor compliance with therapy which may significantly enhance VI progression and severity^28^.

Though level of income and marital status have been found to be significantly associated with VI among elders, this was not found in this study^18^, ^29^, ^30^ These studies that were done in Iran and India attributed these findings to probable lack of a support system and thus reduced access to eye care services. The findings in our study could be due to the strong support systems in the African culture where elders are economically and socially taken care of by their children or grandchildren, so the elders' level of income and marital status may not necessarily influence their access to care^31^. Our findings are comparable to a study done among an elderly population in Taiwan where marital status was not statistically associated with VI^12^, ^32^.

The study has some limitations. This was a cross-sectional study that we couldn't establish temporal associations. The study relied on self-report of information from the study participants, hence there could be information bias since some elders may not honestly disclose some information, for example, level of education, marital status. This was a hospital-based study; hence the findings may not be generalizable to the entire general population.

## Conclusion

The prevalence of VI among the elderly in Mulago hospital is high with the commonest causes being treatable conditions which include cataracts, refractive errors, glaucoma, corneal opacities and over 76% of the causes can be treated by cataract surgery and correction of refractive errors. Age, history of diabetes mellitus, history of hypertension, education, presence of cataract at examination were found to be associated with VI.

We recommend that the elderly with diabetes and hypertension should undergo regular eye examination to detect and manage the causes of VI. Measures to improve the cataract surgical rate in Uganda are needed to address the high prevalence of cataracts.

## List of abbreviations

aPR: adjusted Prevalence Ratio
NCDs: Non-communicable diseases
COVID: Corona virus disease
VI: Visual Impairment
V/A: Visual Acuity
HIV: Human Immunodeficiency Virus
DM: Diabetes mellitus

## Declarations

### Data Availability

The datasets used and analyzed during this study are available from the corresponding author on reasonable request.
